# A Rare Cause of a Scrotal Abscess due to the Symbiotic Infection of *Gardnerella vaginalis* and *Prevotella bivia* in an Adult Male

**DOI:** 10.3390/pathogens9020093

**Published:** 2020-02-01

**Authors:** Anthony Bekasiak, Fabian Dammann, Claudia Nader

**Affiliations:** 1 Department of Medicine, St. Elizabeth’s Medical Center, Boston, MA 02135, USA; anthony.bekasiak@steward.org (A.B.); fabian.dammann@steward.org (F.D.); 2 Tufts University School of Medicine, Boston, MA 02111, USA; 3 Division of Infectious Disease, St. Elizabeth’s Medical Center, Boston, MA 02135, USA

**Keywords:** *Gardnerella vaginalis*, bacterial vaginosis, *Prevotella bivia*, scrotal abscess, sexually transmitted infections, male genitourinary infection

## Abstract

*Gardnerella vaginalis* (*G. vaginalis*) is the major bacteria detected in women with bacterial vaginosis (BV). *Prevotella bivia* (*P. bivia*) has been demonstrated to show a symbiotic relationship with *G. vaginalis*. Some men have been shown to be colonized with *G. vaginalis* in their urogenital or anorectal tracts, however genitourinary infections in males, including balanitis and urethritis, due to this organism appear to be much less common. In this report, we summarize previous cases of men with *G. vaginalis* infection, and we present a rare and unusual case of a unilateral scrotal abscess caused *by G. vaginalis* in co-infection with *P. bivia*.

## 1. Introduction

A 27-year-old male with medical history significant only for morbid obesity with a Body Mass Index (BMI) of 43.9 presented to the emergency room with a three-day history of worsening right scrotal swelling and pain. The pain was non-radiating, dull, and severe in nature. He endorsed subjective fevers and chills, but had no urinary symptoms such as dysuria, hematuria, or frequency. He denied any trauma to the urogenital region. The patient was heterosexual with history of two sexual partners, with his most recent sexual encounter three months prior to his presentation during which a condom was used for barrier precaution. He did not know whether the sexual partner had bacterial vaginosis (BV) or was colonized with these bacteria. He denied any prior history of sexually transmitted infections or Human Immunodeficiency Virus (HIV). 

Upon presentation, he was afebrile with stable vital signs. Genitourinary examination revealed a circumcised penis without any lesions or discharge or evidence of phimosis. The scrotum on the right side appeared erythematous, swollen, and tender, suggestive of a cellulitis, with a large fluctuant palpable mass, but no signs of induration. Blood work was pertinent for a White Blood Cell (WBC) count of 16.1 × 10.3/µL, with 77% neutrophils with left shift. Urinalysis showed <1 high-power field (HPF) WBCs with negative leukocyte esterase and nitrite. Urine nucleic acid amplification test (NAAT) was negative for the presence of pathogen DNA of trichomonas, gonorrhea, and chlamydia. A scrotal ultrasound revealed normal echotexture in both testes without focal abnormalities and no evidence of hydroceles or varicoceles. Arterial and venous outflow was normal. Marked thickening of the skin of the scrotum was noted with echogenic inflamed fat with hyperemia more noticeable on the right. An echogenic collection seen adjacent to the right hemiscrotum with a deeper component measuring up to 4.9 × 2 cm was visualized. A Computed Tomography (CT) of the pelvis with intravenous (IV) contrast was subsequently obtained, which did not reveal any radiographic feature suggestive of Fournier’s gangrene. He was initiated empirically on IV ceftriaxone 1 g daily. He underwent a bedside incision and drainage of the abscess. Purulent material was noted and was sent to microbiology. 

Gram stain revealed many polymorphonuclear cells, few Gram-positive cocci in pairs, and scarce Gram-negative rods. The abscess specimen was plated on sheep blood agar, chocolate agar, and MacConkey agar for aerobic incubation, and was incubated at 37 °C in 5% carbon dioxide. The specimen was also plated on an anaerobic blood agar enriched with vitamin K, an anaerobic phenylethyl alcohol agar, and in thioglycollate broth for anaerobic culture, and incubated at 37 °C in an anaerobic atmosphere. After 72 hours of incubation, small dry colonies with alpha-hemolysis were observed on both the sheep blood and chocolate agars. Catalase test was negative. VITEK 2 system (BioMérieux, Cambridge, MA, USA) was used for rapid bacterial identification, and the organism was identified as *Gardnerella vaginalis* with the VITEK 2 GP ID card. Anaerobic growth was noted after 3 days in broth only. VITEK 2 system (BioMérieux) identified the organism as *Prevotella bivia* with the VITEK 2 ANC ID card. Fungal cultures were negative. The patient had clinical response to the empiric ceftriaxone initiated at presentation, and his WBC down trended to normal range. He was discharged home on cephalexin 500 mg p.o. four times a day, and metronidazole 500 mg three times a day for a total of fourteen days, with instruction to follow up with his primary care physician in one week for repeat scrotal ultrasound and at the wound center to ensure proper wound care and appropriate wound healing. Regrettably, the patient had personal commitments obliging him to leave the state, thus, he was lost for any clinical follow-up. Fortunately, his contact information remained the same, and he was telephoned two months later, with the patient reporting complete resolution of his symptoms. 

## 2. Discussion

*G. vaginalis* is typically associated with BV in women, which is the most common cause of vaginal discharge [1]. BV is characterized by loss of lactic acid-producing *Lactobacillus* spp. that compose the normal vaginal flora, with an increase in facultative and strict anaerobic bacteria, including *G. vaginalis*, *Prevotella* spp., *Atopobium vaginae*, *Sneathia* spp., and other bacterial vaginosis-associated bacteria (BVAB). Recent findings by Muzny et al. show that despite the high abundance of *G. vaginalis* and *P. bivia* in women with BV, neither induced a robust inflammatory response from vaginal epithelial cells, suggesting that these pathogens are early colonizers evading the immune system while establishing the BV biofilm. As a result, secondary colonizers, including *A. vaginae, Sneathia* spp., and other BVAB, act as more potent stimulators of the hos-immune response to BV, leading to the signs, symptoms, and its adverse effects [2]. The treatment of BV in symptomatic women has been established with metronidazole or clindamycin. *G. vaginalis* has been reported to cause complications following delivery or pelvic surgery, such as preterm rupture of membranes, chorioamnionitis, and postpartum fever [3,4,5]. Disease due to *G. vaginalis* in men has been reported but is very uncommon.

### 2.1. Sexual Transmission of Gardnerella vaginalis

In 1953, an organism presumed to be *G. vaginalis* was isolated by Leopold from cultures of urethral swabs and urine specimens from men suffering from urethritis or prostatitis [6]. In 1955, *G. vaginalis* was then further studied by Gardner and Dukes, who found that *Gardnerella* was isolated from the urethras of 86% of husbands of women with BV [7]. In 1984, Piot et al. worked on biotyping *G. vaginalis* and found that biotypes isolated from vaginal cultures of women with BV and from the urethras of their sexual partners were identical more than 90% of the time when sampled within 24 hours; this study further demonstrates the fact that *G. vaginalis* can be sexually transmitted [8]. In the past, it was reported that up to 7%–11% of men had *G. vaginalis* as part of their urogenital or anorectal flora, leading to the possibility of urinary tract colonization and infection [9]. In a cohort study in Sweden of male attendees of a sexually transmitted infection (STI) clinic, urethral carriage of *G. vaginalis* was noted to be 4.5% (10 out of 309) [10]. In uncircumcised men, *G. vaginalis* more frequently colonizes, and is easier to isolate from the glans penis and prepuce than from the urethra, where the organism can easily be eradicated by micturition [11]. Moreover, semen or the prostate gland appear to be possible reservoirs for *G. vaginalis*. A report of an otherwise healthy male with no clinical features to suggest infection presented to an STI clinic, as his female sexual partner had recurring BV infections despite metronidazole treatment. His semen was analyzed, yielding a growth of *G. vaginalis*, and both partners received metronidazole, with resolution of the female’s recurrent BV [12]. In a study by Lam et al., *G. vaginalis* was recovered from 4 out of 12 semen samples, as well 4 out of 7 urethral samples [13]. Semen specimen growth of *G. vaginalis* was also noted in three infertility clinics. One clinic noted that 19% of isolated microorganisms from 36 semen samples were identified as *G. vaginalis* [14]. The second clinic isolated 552 microorganisms from 182 males, 52 out of which were *G. vaginalis* (9.6%) [15]. In addition, when semen samples were obtained with at least 3 days of sexual abstinence, 22 out of 38 semen samples (58%) isolated *G. vaginalis*, raising the possibility of male colonization. There was no association between the isolation of *G. vaginalis* and the sperm count [16]. 

Heterosexual contact, as in female-to-male transmission, appears to enhance male carriage of *G. vaginalis*, as documented by Holst. BV-associated organisms (*Mobiluncus mulieris, *M. curtisii**, and *G. vaginalis*) were isolated in this study from male consorts of women with BV, then after two weeks of consistent condom use, the isolates of the BV-associated organisms disappeared [17]. Similarly, in a later study suggestive for female-to-male transmission, *G. vaginalis* carriage by men was closely linked to lack of condom use [18]. Sexual behavior-related characteristics have also been associated with BV. These include young age at coitarche, life-time number of sex partners, recent history of multiple sex partners, and a recent history of a new sex partner. However, what classifies these factors as high-risk sexual behavior that can lead to BV infections appears inconsistent [19].

### 2.2. Gardnerella vaginalis Male Infections

A review of the literature performed via PubMed database using the Medical Subject Headings (MeSH) of “*Gardnerella vaginalis*” and “male” identified 21 cases of *G. vaginalis* adult male infections published in individual case reports and case series (Table 1). In brief, patients presented with ages between 19 and 78 years, with an average age of 47 years.

While balanitis is usually caused by *Candida* organisms, anaerobes with mixed species, including *G. vaginalis*, were found to be the culprit in some cases [20]. Bacteremia due to *G. vaginalis* has been reported in men with immunosuppression, chronic alcohol use disorder, urethral stenting, meatal strictures, infected urinoma, prostate adenomas, or transurethral prostatectomy [21,22,23,24,25,26,27,28,29,30]. In two otherwise healthy patients, *G. vaginalis* bacteremia was found in a male presenting only with urethritis, raising the question of whether *G. vaginalis* is overlooked, and another with only a positive urine culture [31,32]. Other males with no significant medical histories were found with either urolithiasis or nephrolithiasis leading to pyelonephritis and *G. vaginalis* bacteremia [33,34]. Though limited in detail, a male with an indwelling catheter for over six hours was reported to develop *G. vaginalis* infection [30]. Lastly, another healthy male was found with pyelonephritis associated with *G. vaginalis* septicemia complicated by infective endocarditis and septic emboli to the kidney and brain [35]. Of note, 7 of the 21 cases note some element of prostatic disease, supporting the hypothesis of the prostate as a possible reservoir for *G. vaginalis* [21,25,27,29,30,31].

### 2.3. Prevotella bivia

*Prevotella* spp. are part of the cervical flora and are known to cause pelvic inflammatory disease along with perirectal abscesses [36,37]. In up to 40% of healthy women and in a higher proportion of those with BV, *Prevotella* DNA has been detected in vaginal epithelia [38]. They are Gram-negative anaerobic rods that are related to *Bacteroides* spp.; however, they are not able to grow in the presence of bile and are unable to hydrolyze esculin. They are usually susceptible to clindamycin, metronidazole, imipenem, and amoxicillin/clavulanate [39]. The presence of *Prevotella* in men has also been related by some to BV in their sexual partners [40]. *P. bivia* infection has been reported to cause penile abscesses in concomitant infection with *Streptococcus constellatus*, in which case treatment involved surgical drainage and antibiotic treatment initially with IV vancomycin and clindamycin, and then oral amoxicillin/clavulanate [41].

A commensal relationship between *G. vaginalis* and *P. bivia* has been proposed, with ammonia flow as the mechanism to support the hypothesis. In a study by Pybus et al., strains of *P. bivia* and *G. vaginalis* were studied for nutrient substrate utilization. Ammonia produced by *P. bivia* stimulated *G. vaginalis* growth, and amino acids produced by *G. vaginalis* enhanced *P. bivia* growth [42]. One prospective study investigating the induction of incident BV via daily vaginal specimen collection showed *P. bivia* had a higher mean relative abundance among cases prior to incident BV, suggesting a synergy between *P. bivia* and *G. vaginalis* prior to a BV infection [43].

## 3. Case Discussion

Our patient developed a unilateral scrotal abscess caused by *G. vaginalis* and *P. bivia*. A scrotal abscess may result from the extension of epididymo-orchitis, rupture of testicular abscess, spread of intra-abdominal infection such as appendicitis via patent process vaginalis, a pathological urethro-ejaculatory duct reflux, a hematogenous spread of a systemic infection, or idiopathic process [44]. More superficially, scrotal abscess can result from the spread of cellulitis or an infected hair follicle. 

Our patient became colonized with *G. vaginalis* after acquiring it sexually from one, or both, of his female partners, and was likely harboring it in a seminal or prostatic reservoir. He then developed a scrotal abscess, likely as a result of a minor skin trauma (such as shaving or scratching), causing a skin and soft tissue infection, that spread to the scrotum, or a subclinical urethritis/epididymo-orchitis that extended to the scrotum.

The treatment of *G. vaginalis* in symptomatic women with BV has been established with metronidazole or clindamycin. However, treatment of this infection has not been well studied in men. Since the patient had clinical improvement with IV ceftriaxone, we opted to continue with a beta-lactam upon discharge and add metronidazole for 14 days. 

## 4. Conclusions

We present a case of an adult male with morbid obesity who developed a unilateral scrotal abscess caused by *G. vaginalis* and *P. bivia*. In a review of literature, including several other reported cases of *G. vaginalis*, this case appears to be the first case report of such a clinical presentation. This case also demonstrates that *G. vaginalis* with the symbiotic infection of *P. bivia* may be an occasional cause of genitourinary abscess formation in both males and females, and its growth should not be neglected. Given the sexual transmission of *G. vaginalis*, the growth of this bacteria in a male should be considered as a pathogen in males presenting with clinical urogenital disease and as a reservoir for recurrent bacterial vaginosis in his female partner.

## Figures and Tables

**Table 1:** Reported cases of *G. vaginalis* infections in male patients.

Year	Corresponding Author and Journal [Reference]	Case Description	Culture Site	Culture Results	Antibiotic Therapy Duration in Days (If Reported)
1978	Abercrombie *Lancet* [32]	20-year-old healthy male developed UTI.	Urine	*G. vaginalis*	Doxycycline (n/a)
1978	Patrick *Lancet* [27]	57-year-old male developed *G. vaginalis* bacteremia following TURP.	Blood	*G. vaginalis*	TMP-SMX (n/a)
1981	Finkelhor *New England Journal of Medicine* [26]	37-year-old male with renal transplant (on azathioprine and prednisone) developed ascending *G. vaginalis* UTI and perinephric abscess.	I. Urine II. Perinephric abscess	I. *G. vaginalis* II. *S. epidermidis* and *Enterobacter* sp.	Doxycycline (n/a)
1986	Burdge *Sexually Transmitted Diseases* [20]	29-year-old male with gonorrhea treated 2 years prior developed urethral discharge, erythema of glans penis, odor, and slight dysuria of 6-month duration.	Urethra	*G. vaginalis*, *Viridans streptococci*, and *S. epidermidis*	Metronidazole 500 mg BID (7 days)
1986	Burdge *Sexually Transmitted Diseases* [20]	31-year old healthy male developed urethral discharge, pruritis of prepuce, erythema of glans penis, and odor of 9-month duration. Multiple recurrences and treatments followed suit.	Urethra	*G. vaginalis*	I. Metronidazole 2 g p.o. once and tetracycline 500 mg QID (7 days) II. Erythromycin 500 mg QID (14 days) III. Metronidazole (wife treated as well) (7 days) IV. Erythromycin 500 mg QID (two 14-day courses) V. Clindamycin 450 mg TID (10 days)
1986	Burdge *Sexually Transmitted Diseases* [20]	42-year-old male with gonorrhea treated 7 and 12 years prior developed 3-year history of intermittent urethral discharge with odor.	Urethra	*G. vaginalis*	I. Metronidazole 500 mg BID (7 days) (wife treated also) II. Clindamycin 450 mg TID (two 10-day courses)
1986	Wilson *British Medical Journal* [24]	19-year-old male with meatal stricture attributed to childhood circumcision developed dysuria and frequency with *G. vaginalis* septicemia.	Blood, urine	*G. vaginalis*	(n/a)
1987	Harper *Journal of Clinical Pathology* [29]	60-year-old male developed *G. vaginalis* septicemia following TURP.	Blood, urine	*G. vaginalis*	Gentamicin (n/a)
1989	Sturm *Journal of Infection* [30]	53-year-old male developed infected urinoma due to *G. vaginalis*.	Urine	*G. vaginalis*	Amoxicillin and metronidazole (n/a) Underwent right-sided nephrectomy
1989	Sturm *Journal of Infection* [30]	67-year-old male presented with prostatitis.	Urine	*G. vaginalis*	(n/a)
1989	Sturm *Journal of Infection* [30]	61-year-old male noted with prostatitis.	Urine	*G. vaginalis*	(n/a)
1989	Sturm *Journal of Infection* [30]	72-year-old male developed cystitis after indwelling catheter of +6-hour duration	Urine	*G. vaginalis*	(n/a)
1989	Legrand *Journal of Clinical Microbiology* [23]	41-year-old male with chronic alcohol use disorder developed *G. vaginalis* bacteremia and pulmonary abscess.	I. Sputum II. Blood III. Pleural fluid	I. Non-hemolytic *Streptococci* and *Corynebacterium-like* organisms (reported as contaminated flora) II. *G. vaginalis* (from second lot) and *Strep. milleri* (from third lot) III. *Bacteroides oralis*, *Peptostrep.* sp., *Peptococcus* sp., *Veillonella* sp., *H. parainfluenzae, Strep. milleri*	Ceftazidime, penicillin, minocycline, metronidazole, clindamycin and ampicillin; chloramphenicol injection into the pleural space (19 days) (deceased during hospital course)
1990	Denoyel *Journal of Infectious Disease* [25]	65-year-old male with prostate adenoma developed *G. vaginalis* bacteremia.	Blood	*G. vaginalis*	Gentamicin and amoxicillin (n/a)
1997	Bastida *European Journal of Clinical Microbiology & Infectious Disease* [21]	78-year-old male with history of lung resection for lung malignancy, chronic alcohol use disorder, chronic prostatic symptoms, and recurrent UTI developed *G. vaginalis* bacteremia.	Blood	*G. vaginalis*	Cefonicid IV (10 days)
2005	Calvert *Journal of Infectious Disease* [22]	50-year-old male with chronic alcohol use disorder and tobacco dependency developed *G. vaginalis* sepsis with perinephric abscess and empyema.	Blood, perinephric abscess, pleural fluid	*G. vaginalis*	Benzylpenicillin, cefuroxime, and metronidazole; discharged on oral metronidazole and amoxicillin (79 days total)
2008	Lagace *Journal of Clinical Microbiology* [33]	41-year-old healthy male developed *G. vaginalis* bacteremia.	Blood	*G. vaginalis*	Ciprofloxacin IV (10 days)
2010	Yoon *International Journal of STD & AIDS* [35]	39-year old male with diabetes mellitus developed *G. vaginalis* septicemia with pyelonephritis, infective endocarditis, and septic emboli in the kidney and brain.	Blood	*G. vaginalis*	IV metronidazole, ceftriaxone, and oral erythromycin, discharged on p.o. metronidazole (96 days); then aortic valvuloplasty
2013	Alidjinou *Médicine et Maladies Infectieuses* [28]	61-year-old male with sigmoid colon cancer with urethral stenting for tumor-related hydronephrosis developed *G. vaginalis* bacteremia.	Blood	*G. vaginalis*	Amikacin, ceftriaxone + metronidazole (10 days)
2015	Babics *Médicine et Maladies Infectieuses* [31]	36-year-old male with history of recurrent UTIs developed *G. vaginalis* bacteremia.	Blood, Urine	*G. vaginalis*	Azithromycin (14 days)
2017	Pritchard *Infectious Diseases in Clinical Practice* [34]	36-year old male with nephrolithiasis developed pyelonephritis and *G. vaginalis* bacteremia.	Blood, Urine	*G. vaginalis*	Metronidazole (14 days)

Abbreviations: BID: twice a day; TID: three times a day; QID: four times a day; TMP-SMX: trimethoprim-sulfamethoxazole; UTI: urinary tract infection; TURP: transurethral prostatectomy; n/a = not available; sp.: species.
